# Exploring the experiences of sonography students with simulation‐based learning: A perspective from South Africa

**DOI:** 10.1002/jmrs.814

**Published:** 2024-08-12

**Authors:** Geordean Schwartz, Kathleen Naidoo, Ferial Isaacs

**Affiliations:** ^1^ Cape Peninsula University of Technology—Bellville Campus Ringgold Standard Institution—Medical Imaging and Therapeutic Sciences Bellville Western Cape South Africa

**Keywords:** simulation‐based learning (SBL), sonography education, student experiences

## Abstract

**Introduction:**

Simulation‐based learning (SBL) is widely used in healthcare education to provide a safe environment for students to practice clinical scenarios without causing patient harm. While established in developed countries, SBL's implementation is new in South Africa; there is a lack of research addressing sonography students' experiences. This study aimed to explore and describe the experiences of Bachelor of Science (BSc) second‐year sonography students using SBL for clinical training at a local University of Technology (UoT).

**Method:**

An exploratory, qualitative and descriptive research study was conducted in 2020, with virtual one‐on‐one interviews due to COVID‐19 restrictions. Eight BSc second‐year sonography students, who had undergone SBL in their first year, participated. Data saturation was achieved, and interviews were audio recorded and transcribed verbatim.

**Results:**

Thematic analysis revealed three themes: (1) Enhancing preparedness for the clinical environment, (2) Limitations of the tissue‐equivalent phantom and (3) Suggestions for improving simulation. While students expressed positive feedback and enjoyment of the simulation tool, they also highlighted limitations, such as unrealistic representations of real patient scanning conditions.

**Conclusion:**

This study provides valuable insights into sonography students' experiences with SBL. Positive influence of SBL on clinical training was observed. To enhance SBL for future sonography students, consideration for high‐fidelity simulators with advanced software is recommended. Funding options to invest in such simulators should be explored by radiography educators to promote more realistic training experiences.

## Graphical abstract

This study aimed to explore and describe the experiences of BSc second‐year sonography students using simulation‐based learning (SBL) as a form of clinical training prior to patient interaction at a University of Technology (UoT) in South Africa. Thematic analysis of the data revealed three themes: (1) Enhancing preparedness for the clinical environment, (2) Limitations of the tissue‐equivalent phantom and (3) Suggestions for improving simulation learning.

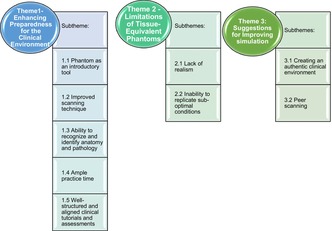

## Introduction

The integration of ultrasound simulators in medical education has advanced the implementation of simulation‐based learning (SBL).^1^ SBL is recognised as an effective educational approach that replicates clinical scenarios to develop critical thinking skills within a safe and controlled environment, ensuring patient safety.^2^, ^3^ Critical thinking skills necessary for sonographers are essential higher order cognitive functions in Bloom's taxonomy of learning objectives.^4^ Simulation has been widely adopted in various medical disciplines, including sonography, nursing, emergency medicine, anaesthesia, surgery and radiography, to enhance clinical preparedness.^5^, ^6^ It provides a secure and controlled environment to replicate lifelike situations and facilitate learning.^5^, ^7^

Simulation‐based learning offers significant advantages, allowing sonography students to practice ultrasound examinations at their own pace and providing opportunities for repeated scans to enhance sonographic skills development.^5^ Students can apply theory into practice and develop confidence through simulated interactions before performing the same examinations on actual patients.^5^, ^7^ Mastery of essential components in ultrasound, including knobology (knowledge of ultrasound machine settings and functions), image acquisition and interpretation (anatomical landmarks, organ location, biometric measurements, shape, outline, composition and echogenicity) can all be achieved through SBL before patient interactions.^8^

While SBL offers numerous benefits, it is important to acknowledge its limitations in fully replicating actual patients or live volunteers, as simulators only provide lifelike imitations.^1^ Different types of ultrasound simulators exist, including low‐fidelity and high‐fidelity simulators, each with varying degrees of realism and cost.^9^ High‐fidelity simulators employ virtual or computer‐based simulations, incorporating features such as transducer tracking systems and dynamic virtual sonograms, offering greater realism and functionality as compared to low‐fidelity simulators.^8^, ^10^ These simulators can simulate anatomical variants, clinical scenarios and ultrasound pathology in 2D, providing a comprehensive learning experience for students.^7^, ^10^ On the other hand, low‐fidelity simulators, such as most tissue‐equivalent phantoms, offer a cost‐effective alternative for practicing basic technical skills and mirroring clinical scenarios, despite their limited realism.^9^, ^11^, ^12^, ^13^

In South Africa, workplace Learning (WPL) is mandatory for sonography students to obtain on‐the‐job experiential training at accredited hospitals. This is aligned with the requirements of the Health Professions Council of South Africa (HPCSA). In the past, sonography students were enrolled at the university for a 3‐year National Diploma (N‐Dip) programme. In order to gain clinical competency and skills through WPL, the students were assigned to hospitals/clinical facilities, accredited for clinical training, from the first to third year. Minimal clinical competency was achieved in the first year, leaving the remaining 2 years for students to achieve clinical competency. The number of students enrolled was determined by the number of clinical staff available for clinical supervision at the accredited hospitals/clinical facilities. The ratio 1:1 (staff to student) is HPCSA's regulation for ultrasound training in South Africa. This limitation on the student enrolment and placement in clinical facilities is thus a challenge especially as ultrasound is considered a scarce skill in South Africa.^14^, ^15^

The current 4‐year BSc Diagnostic Ultrasound degree was introduced in 2014 at a University of Technology in the Western Cape province of South Africa. The new degree was designed to incorporate intensive SBL at a well‐resourced clinical laboratory at the university campus, preparing the students with the necessary knowledge and practical skills during the first year before entering the clinical facilities to scan patients in the second year. The aim was thus to enrol more students for the 4‐year degree compared to the 3‐year diploma, establish a more effective WPL period of 3 years, produce a well‐rounded graduate with clinical competency in most fields in diagnostic ultrasound and thus enhance diagnostic ultrasound service delivery in South Africa.

**Figure 1:**
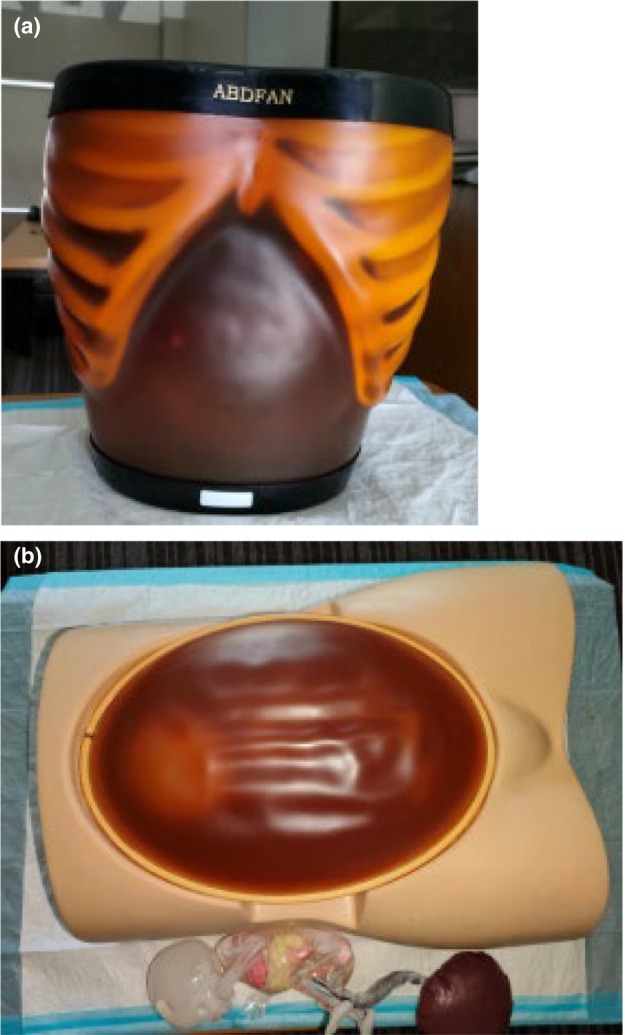
Ultrasound training phantoms (a) Kyoto Kagaku ABDFAN, abdomen and (b) SPACE FAN‐ST, obstetric. (a) Kyoto Kagaku ABDFAN abdominal ultrasound training phantom (Clinical skills lab; 2020). (b) SPACE FAN‐ST obstetric ultrasound examination phantom (Clinical skills lab; 2020).

Simulation‐based learning was key to preparing the students for WPL, that is, scanning patients. To provide effective SBL opportunities, funding was allocated to acquire tissue‐equivalent ultrasound phantoms (‘mannikins’). Two high‐fidelity tissue‐equivalent phantoms, namely the Kyoto Kagaku ABDFAN abdominal ultrasound training phantom and the SPACE FAN‐ST foetus ultrasound examination phantom, were procured to support the training needs of first‐year sonography students (refer to Fig. 1). These tissue equivalent phantoms are considered high fidelity because the one contains abdominal pathology, and the obstetric simulator can be adjusted to accommodate breech, cephalic and spine up/down foetal positions. The phantoms facilitated the practice of novice sonography scanning during the student's first year, primarily at the university clinical skills laboratory. From the second to the fourth year, students were placed at accredited training hospitals/healthcare facilities for WPL.

The aim of this study was to explore and describe sonography students' experience using SBL within the South African context.

## Methods

### Study design

A qualitative, exploratory, descriptive and contextual approach was employed to conduct this research study. Individual semi‐structured interviews were conducted with BSc second‐year sonography students to explore and describe their experiences of using simulation before patient interaction (refer to Table 1). The framework for the interview questions was adapted from Gibbs,^1^ and permission was granted. The researcher acted as a neutral facilitator for the interviews, not assuming the role of a sonographer or lecturer, which assisted in establishing a comfortable atmosphere for the participants. The bracketing process was implemented to minimise the influence of researchers' feelings and opinions on data collection.^16^ To ensure transparency and minimise bias, the researcher maintained a reflexive journal throughout the study.

**Table 1:** Semi‐structured interview questions.

**The following semi‐structured questions were used as a framework to guide the interviews**
*Question 1*
I would like you to reflect on your experiences when you were trained using the tissue‐equivalent phantoms
*Question 2*
Now that you are scanning real patients, do you feel the simulator had any impact on your learning?
*Question 3*
If yes, please explain how and give some examples
*Question 4*
Did the simulators in any way improve specific areas such as: scanning technique; use of the equipment controls; your hand/eye coordination; recognising normal anatomy or pathology?
*Question 5*
What role did the simulator have, if any, regarding your learning experience?
*Question 6*
Do you think the simulators were appropriately used in your learning and for the formative clinical assessments? Please explain your answer
*Question 7*
You are scanning real patients now. Could you reflect on the advantages and disadvantages, of any of the simulator interactions and scanning real patients?

This research included BSc second‐year sonography students registered with the Health Professions Council of South Africa (HPCSA) at a University of Technology in the Western Cape. Purposive sampling was used to select participants who had experienced SBL in their first year of study. The selection of BSc second‐year students as participants in this study was based on their completion of the first year of SBL and their ability to provide valuable insights into their experiences.

As part of the SBL using the simulators, BSc Diagnostic Ultrasound first‐year students had abdominal and obstetric formal lectures and had written tests. All the formal lectures were followed by clinical instruction, providing one‐on‐one and group (maximum of four students in a group) simulation instruction sessions. The clinical lecturer provided demonstrations, and these sessions were guided by an assessment rubric and criterion (refer to Appendix S1 section) made available to students. Thereafter, the students could practice for months before being formally assessed on the simulators. The students had one mock clinical assessment in preparation for the formal final clinical assessment scheduled close to the end of the first year, and students received immediate feedback after each clinical assessment. The students were unaware of the level of fidelity of the simulators used, whether low or high fidelity during the research study.

To the best of the researcher's knowledge, this study is the first to explore and describe the experiences and perceptions of sonography students using simulation as a form of clinical training before interacting with patients, specifically within the South African context, since the introduction of simulation in sonography at the university.

Eight BSc Diagnostic Ultrasound second‐year sonography students voluntarily participated in this study, comprising of five females and three males, with ages ranging from 19 to 35 years. Semi‐structured interviews were conducted virtually using WhatsApp video calls due to COVID‐19 lockdown restrictions in August and September 2020. The interviews were conducted, and data saturation was reached after the fifth interview; however, since eight students volunteered, the interviews continued so as not to exclude anyone. The interviews, ranging from 20 to 40 min, were audio recorded and transcribed verbatim after obtaining written consent from the participants. To ensure credibility of the data collection process, debriefing sessions with research supervisors were conducted after each interview, and an audit trail consisting of field notes and a reflexive journal was maintained.

### Trustworthiness

The trustworthiness of the study was ensured by following the criteria of credibility, transferability, dependability and confirmability. Triangulation of data sources and methods was employed to enhance credibility.^17^ Analytic summaries, verbatim quotes and a thorough description of the research setting and data population were provided to facilitate transferability. Dependability was achieved through well‐organised methods and detailed audit trials. Confirmability was established by conducting a confirmability audit, including audio tape recordings, coding details and field notes. Additionally, the themes and categories derived from the data were shared with the participants to ensure their agreement and reflection on their experiences.^18^

### Data analysis

Thematic analysis following Braun and Clarke's method was employed to analyse the transcribed data using six steps:^19^, ^20^, ^21^

1. Reading and re‐reading the transcribed data.
2. Identifying phrases and coding or labelling the data.
3. Processing and combining all repetitive phrases looking for patterns of association.
4. Review phrases and codes to form a potential theme.
5. Defining each possible theme and subthemes.
6. Write a report clearly defining each of the themes and subthemes.

The researcher immersed himself in the data by reading and re‐reading the transcriptions and listening to the audio recordings. Preliminary codes in the form of keywords and phrases were allocated to identify recurring patterns. Themes and categories were developed through an inductive reasoning process.

### Ethical considerations

Ethical permission was obtained from the research site ethics committee (CPUT/REC2020/H10). The study adhered to ethical principles such as beneficence, non‐maleficence, autonomy and justice. Risks to participants were minimised, and data anonymisation and secure storage practices were implemented to ensure confidentiality and data protection. Participants were informed of their right to withdraw from the study at any time, and their identities and personal identifiers were anonymised.

## Results

**Figure 2:**
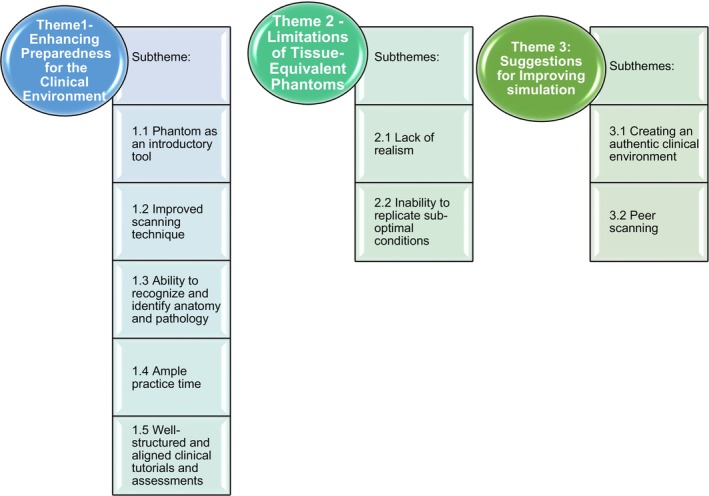
Themes and subthemes.

The data collected led to the identification of three themes, which centred on the participants' authentic narratives and shared experiences (refer to Fig. 2). The themes included are as follows:

- Theme 1: Enhancing preparedness for the clinical environment,
- Theme 2: Limitations of the tissue equivalent phantom and
- Theme 3: Suggestions for improving simulation.

The themes are presented along with conceptualisation with the literature.

### Theme 1: Enhancing preparedness for the clinical environment

This theme explored participants' stories and experiences regarding the use of simulation as an introduction to the clinical environment. The participants acknowledged the benefits of using tissue‐equivalent phantoms for improving their understanding of abdominal and obstetric ultrasound anatomy and developing their scanning technique. The participants expressed gratitude for the opportunity to gain confidence and mastery through ample scanning time during simulation practice sessions. Participants perceived the use of tissue‐equivalent phantoms in SBL as an effective means of introducing ultrasound concepts. This approach alleviated feelings of anxiety and nervousness by allowing participants to practice before interacting with actual patients. The phantoms provided a visual representation of internal organs on ultrasound, thereby facilitating the transition to working with real patients. From the collected data, participants emphasised the valuable role of the phantom in acquiring clinical skills and enhancing their scanning technique in preparation for the clinical environment. They expressed how the phantom facilitated learning to adjust ultrasound machine settings and manipulate the transducer to obtain diagnostically relevant images. Participants also highlighted feeling more comfortable while practicing their scanning technique on the phantoms. (Refer to participant responses in Table 2).

**Table 2:** Identified themes and participant responses (a) Theme 1—Enhancing preparedness for the clinical environment, (b) Theme 2—Limitations of the tissue equivalent phantom, (c) Theme 3—Suggestions for improving simulation.

**a) Theme 1—Enhancing preparedness for the clinical environment**
Participant 1: ‘Using the clinical phantom, it was something new… I don't think scanning is easy. But I think it was good to see how all the organs look on ultrasound prior to we move to real patients’
Participant 2: ‘I think like we, in the phantoms we can repeat mistakes like several times without getting any pressure… in the clinicals like there's that pressure, like you can't repeat mistakes like for several times’
Participant 5: ‘Ja (sic) definitely. Scanning technique, definitely. How to manipulate the transducer, the machine, how to adjust the settings on there, ja (sic)’
Participant 6: ‘I learned how to use focal zones and all the frequencies to adjust it according to the patient’
Participant 7: ‘It kinda (sic) gives you confidence to get yourself oriented very well on how to use the probe and where to get the things on the phantom’
Participant 8: ‘As I said like (sic) I can distinguish between a pathological thing and a normal thing… with the phantom it really helps in making the learning experience… learning everything…… so also like (sic) be able to incorporate that into the clinical thing’
**b) Theme 2—Limitations of the tissue equivalent phantom**
Participant 3: ‘The phantom doesn't have breathing… It doesn't give you any movement… echogenicity is different, it even feels different’
Participant 4: ‘I was kind of spoilt by the phantom. When I was about to scan a real patient… I had some struggles but… I got a way to sort that out’
Participant 5: ‘Okay, starting to get so comfortable with scanning the phantom when you haven't scanned a real patient yet. You become so comfortable that you think it's easy, and when you get to the clinical department and you realise that scanning a real patient isn't that easy, then it's like a shock to your body. I think we also need to be exposed maybe in the classroom to suboptimal images so that we can get used to…focus on images that are not of the perfect patient. Maybe it would make it easier for us to identify the anatomy on patients when you get to the department with bowel gas and all those things as well’
Participant 6: ‘Disadvantages were the phantom doesn't have breathing or bowel gas… it doesn't have any blood flow’
Participant 8: ‘Everything was just stationary… it was a bit difficult scanning a real patient’
**c) Theme 3—Suggestions for improving simulation**
Participant 4: ‘They can go to the hospital just to observe, not to scan…be aware what the real patient looks like… during the vacation during June, if ever they can get an opportunity, they can go and can just observe’
Participant 5: ‘I think they need to get phantoms that have more adipose and more gas in it… to try to identify the structures…get used to images that aren't as pretty’
Participant 6: ‘Later in the year that the first‐year students scan on a real patient… just to get the feel of how the real patient's anatomy and everything works’
Participant 7: ‘I think it would really be good… to actually divide ourselves into groups or have a roster where we scan each other… we are more real than the phantom so I think if we were to… scan in that manner… it would be very nice’
Participant 8: ‘We okay, I feel like also the other thing that we should stress on students is on how to do patient care. Especially patient care and how to…greet and stuff (sic) so that even though sometimes we are scanning the patient we should also apply that’

### Theme 2: Limitations of tissue‐equivalent phantoms

This theme explored the challenges encountered by the student cohort while interacting with the tissue‐equivalent phantoms. The participants identified limitations in both the abdominal and obstetric phantoms. The abdominal phantom lacked the pelvic region, including organs such as the urinary bladder and iliac vasculature. On the other hand, the obstetric phantom allowed for changing the foetus's position, but its static nature during scanning did not replicate real obstetric patient scenarios with foetal movement. Some participants felt that the phantom experience, while beneficial, led to difficulties when transitioning to scanning real patients. This theme explored the challenges faced by the student cohort while interacting with the tissue‐equivalent phantoms. The participants highlighted major limitations of the simulators in replicating real patient conditions, such as bowel gas shadowing, cardiac and vascular motion, increased Body Mass Index (BMI) and a moving foetus. Despite the phantoms' usefulness, the lack of realism in the phantoms was evident, as expressed by the participants. (Refer to participant responses in Table 2).

### Theme 3: Suggestions for improving simulation

This theme revolves around the participants' suggestions to enhance the SBL experience. They emphasised the need for better patient care and communication during simulated‐based teaching sessions. Additionally, they expressed a desire to observe clinical staff in action during their first year to better prepare for working in hospitals/clinical facilities and scanning real patients. Participants strongly advocated for clinical simulation experience by scanning student peers or real human models before interacting with actual patients. They suggested that this approach would be more cost‐effective and provide more realistic experiences compared to using the tissue‐equivalent phantoms. (Refer to participant responses in Table 2).

## Discussion

### Theme 1—Enhancing preparedness for the clinical environment

Acquiring clinical skills and mastering ultrasound scanning techniques are essential components of sonography education.^5^, ^7^, ^13^ According to Gibbs^1^, ^5^ and Wells & Goldstein^13^ when simulation phantoms are used as an introduction prior to scanning patients, students feel better prepared for the clinical environment. Simulation learning allows for training to take place in a safe and controlled environment; hence, novice sonography students feeling more relaxed and less pressured. These skills encompass hand–eye coordination, proficient adjustment of ultrasound machine settings based on the tissue being evaluated and accurate manoeuvring of transducers to visualise specific anatomical regions.^5^ The participants expressed that the simulator phantom significantly contributed to the improvement of their scanning technique. Osborn et al.^12^ note that SBL provides practical hands‐on scanning experiences, facilitating the development of advanced psychomotor skills. These skills are subsequently integrated with cognitive abilities to recognise patterns and aid in clinical interpretation and image acquisition.

Participants reported that SBL using the tissue‐equivalent phantoms helped them identify normal anatomy and landmarks, allowing them to be more prepared when entering clinical settings. This finding aligns with Gibbs'^5^ observation that exposure to normal anatomy and pathology through simulation assists students in becoming familiar with anatomical landmarks before imaging real patients. SBL enhanced Australian students' initial experiences to identify ultrasound anatomy and to do pattern recognition prior to scanning patients.^7^ Similarly, the students in this study could better concentrate when scanning the simulator because it was performed in a more relaxed and controlled environment.

Cook et al.^22^ demonstrated a statistically significant improvement in anatomical knowledge among medical students who underwent gynaecological and first‐trimester obstetric ultrasound SBL. Increased practice time with SBL has been associated with improved clinical skills and outcomes among students.^23^ Taylor et al.^24^ explains that SBL should be asynchronous and accessible for students to utilise at a time that suits them best to allow optimal time with the simulator to practice as much as is needed. Hani et al.^25^ expressed cognition that all students are different. Some students may reach a level of competency after one or two ultrasound practice sessions; however, others may need more time to be on par. Gibbs^26^ supports the notion that allowing students ample time for practice enhances their confidence and preparedness for real clinical settings. The participants' narratives align with these findings, indicating the benefits of extended practice time on the phantom in reducing pressure and facilitating skill development.

### Theme 2—Limitations of tissue‐equivalent phantoms

Wells and Goldstein^13^ describe low‐fidelity simulators as being low‐cost, reusable and used to acquire the basic ultrasound clinical skills that students need. Conversely, high‐fidelity simulators have greater realism but are more costly. High‐fidelity simulators can be divided into two categories, namely those that only demonstrate static anatomy and others that can demonstrate dynamic anatomy, for example, replicating a beating heart.^27^ Massoth et al.^11^ maintain that high‐fidelity simulation is not necessarily superior to low‐fidelity simulation and reported that students using high‐fidelity simulators may become overconfident. Furthermore, Toserud et al.^28^ used both low‐fidelity and high‐fidelity simulators to evaluate nursing students' perceptions using simulation as a learning method and concluded that students felt more satisfied when using the low‐fidelity simulator.

The tissue‐equivalent phantoms used in this study (Kyoto Kagaku ABDFAN abdominal ultrasound training phantom and SPACE FAN‐ST foetus ultrasound examination phantom in Fig. 1) are part of a high‐fidelity simulator group that can only demonstrate static anatomy and are lower in cost compared to the more expensive computerised or dynamic anatomy high‐fidelity simulators that are on the market. High‐fidelity simulators that have dynamic anatomy can demonstrate a more realistic or virtual simulation experience with better physiological abilities and a greater degree of complexity in the form of clinical case‐based scenarios.^10^, ^27^, ^28^ In this theme, it was evident that the participants felt that the tissue‐equivalent phantoms lacked realism. The participants expressed a need for more physiological capabilities in the phantoms, such as those found in dynamic or virtual anatomy high‐fidelity phantoms or live human volunteers.

There still exists much debate in the literature on whether high or low‐fidelity simulators are superior. However, when used in the clinical setting, Alinier^24^ opines that both can be used in clinical training because low‐fidelity simulators can provide excellent teaching of foundational skills and high‐fidelity simulators that exhibit greater realism may be used for more complex simulated teaching such as case‐scenario based training in nursing education. While the participants of this study appreciated utilising the tissue‐equivalent phantoms, their stories indicate a need for a combination of high and low fidelity.

### Theme 3—Suggestions for improving simulation

Observation of clinical examinations prior to conducting them independently could aid students to familiarise themselves with the clinical setting, build confidence and observe what is expected of them. Hazell et al.^29^ reported that radiography students in the United Kingdom spend approximately 5 weeks just observing clinical procedures prior to starting to practise actual clinical examinations under strict supervision in the clinical setting. This is in keeping with the suggestion made by the participants of this study. Students are generally more comfortable giving one another feedback during simulation sessions and assisting each other while scanning by actively participating and engaging in experiential learning.^30^ Cho and MacArthur^31^ opine that receiving feedback from multiple peers is more valuable than receiving feedback from one peer or one lecturer/facilitator. This could be the reason why participants suggested scanning their peers as another form of SBL. However, Michael et al.^32^ state that having scan models for patient simulation, as in the case of using sonography student peer models, requires some ethical considerations that include a scan model consent form, Protection of Personal Information Act (POPIA) and data de‐identification, medical history disclosure form and incidental finding referral form in instances where an abnormal finding is detected during a routine simulated session.^33^ Michael et al.^32^ report that practical and clinical skills training activities may have a varied occurrence of between 1.5% and 1.9% having unexpected ultrasound incidental findings that would require a referral from medical schools' skills laboratories to clinicians. This is an important aspect to consider.

This study revealed that the use of tissue‐equivalent phantoms in SBL contributed to increased confidence and comfort levels among students, aligning with prior research supporting the benefits of SBL for enhancing students' readiness for clinical settings. Moreover, this study highlighted SBL as an introductory tool in promoting better preparedness for junior students as they transition to the clinical environment. One major limitation identified in this study was the lack of realism in simulating suboptimal conditions encountered during actual patient scanning. To address this limitation, this study recommended investing in dynamic anatomy high‐fidelity simulators to provide a more realistic and immersive simulation experience. The use of high‐fidelity simulators was found to offer complex and diverse clinical scenarios, facilitating better learning outcomes and preparing students for real‐world scenarios. The COVID‐19 pandemic further highlighted the significance of simulation resources in medical radiation sciences training, prompting the need for continued investment in high‐fidelity phantoms for improved preparedness and learning outcomes.

### Limitations

This study was restricted to a small group of sonography students who were enrolled in one of the three South African tertiary institutions that offer sonography. Additionally, it was limited to one province—the Western Cape. Due to lockdown laws and the COVID‐19 pandemic, all the interviews were conducted virtually rather than in person. One participant found it difficult to conduct the interview because it depended on a reliable wireless network. There were no additional restrictions or difficulties encountered while conducting the research.

## Conclusion

In conclusion, this study supports the value of SBL in sonography education, particularly through the use of tissue‐equivalent phantoms, as a means of enhancing students' confidence and preparedness for the clinical environment. Embracing technological advancements and prioritising investment in simulation resources can lead to long‐term benefits and improved learning outcomes in sonography education. The participants provided valuable suggestions to enhance the SBL experience, by creating a more authentic clinical environment and incorporating simulated peer scanning. These recommendations could lead to improved preparedness and confidence among sonography students before engaging with real patients in the clinical setting. The advantages of SBL highlighted in this study reinforce its importance as an effective educational tool for sonography students.

## Conflict of Interest

The authors declare no conflict of interest.

## Supporting information


**Appendix S1.** Example of Phantom Abdominal assessment rubric for instruction and assessment.

## Data Availability

The data that support the findings of this study are available from the corresponding author upon reasonable request. Due to privacy and ethical considerations, raw data containing sensitive or identifiable information cannot be made publicly available. However, aggregated and anonymized data can be shared with qualified researchers who meet the criteria for access to confidential data. Interested parties may contact the corresponding author at schwartzg@cput.ac.za to request data access and discuss the terms and conditions for data sharing.
