# Supplementary material for: Exploring the experiences of sonography students with simulation‐based learning: A perspective from South Africa
Source: J Med Radiat Sci. 2024 Aug 12;71(4):573–81. doi: 10.1002/jmrs.814 (PMC11638368; doi:10.1002/jmrs.814)
Supplement: Supplementary file 1 — Appendix S1. Example of Phantom Abdominal assessment rubric for instruction and assessment. [file JMRS-71-573-s001.docx]

Supporting information- Example of Phantom Abdominal assessment rubric for instruction and assessment

The clinical instructor reviews the rubric with students before and during the practical demonstration. Afterward, students practice a minimum of 3 months before being assessed.

| **1**. **Scanning technique** | | | | |
| --- | --- | --- | --- | --- |
|  | Good (3) | Fair  (2) | Poor  (1) | Critical error/ Not done (0 or fail) |
| **1.1 Aorta & IVC** |  |  |  |  |
| 1.1.1. Scan the AO and IVC in longitudinal and transverse planes |  |  |  |  |
| 1.1.3. Measure the diameter of the AO and IVC |  |  |  |  |
| 1.1.4. Identify all the AO branches: Celiac artery, SMA and renal arteries |  |  |  |  |
| 1.1.5. Label all the anatomical structures correctly on the ultrasound image |  |  |  |  |
|  |  |  | **Sub-total** | **/12** |
| **Comment:** | | | | |
| **1.2. Liver** |  |  |  |  |
| 1.2.1. Scan the left lobe of liver longitudinal and transverse planes |  |  |  |  |
| 1.2.2. Scan the right lobe of liver in longitudinal and transverse planes |  |  |  |  |
| 1.2.3. Measure the right lobe of liver in longitudinal |  |  |  |  |
| 1.2.4. Compare the echogenicity of the liver to that of the right kidney cortex |  |  |  |  |
| 1.2.5. Scan the hepatic veins with grey-scale and measure |  |  |  |  |
| 1.2.6. Scan the portal vein with grey-scale and measure |  |  |  |  |
|  |  |  | **Sub-total** | **/18** |
| **Comment:** | | | | |

| **1.3. Gall bladder** | | | | |
| --- | --- | --- | --- | --- |
|  | Good (3) | Fair (2) | Poor (1) | Critical error/ Not done (0 or fail) |
| 1.3.1. Scan the gallbladder in longitudinal and transverse planes |  |  |  |  |
| 1.3.2. Measure the length, width and anterior-posterior diameter |  |  |  |  |
| 1.3.3. Measure the gallbladder wall |  |  |  |  |
| 1.3.4. Scan and measure the common bile duct (CBD) |  |  |  |  |
| 1.3.5. Label all the anatomical structures correctly on the ultrasound image |  |  |  |  |
|  |  |  | **Sub-total** | **/15** |
| **Comment:** | | | | |
| **1.4. Right kidney** |  |  |  |  |
| 1.4.1. Scan the right kidney in longitudinal and transverse planes |  |  |  |  |
| 1.4.2. Measure the right kidney in longitudinal and transverse planes |  |  |  |  |
| 1.4.3. Label all the anatomical structures correctly on the ultrasound image |  |  |  |  |
|  |  |  | **Sub-total** | **/9** |
| **Comment:** | | | | |
| **1.5. Left kidney** |  |  |  |  |
| 1.5.1. Scan the left kidney in longitudinal and transverse planes |  |  |  |  |
| 1.5.2. Measure the left kidney in longitudinal and transverse planes |  |  |  |  |
| 1.5.3. Label all the anatomical structures correctly on the ultrasound image |  |  |  |  |
|  |  |  | **Sub-total** | **/9** |
| **Comment:** | | | | |
| **1.6. Spleen** | | | | |
|  | Good (3) | Fair  (2) | Poor  (1) | Critical error/ Not done (0 or fail) |
| 1.6.1. Scan the spleen in longitudinal and transverse planes |  |  |  |  |
| 1.6.2. Measure spleen in longitudinal plane |  |  |  |  |
| 1.6.3. Assess spleen with linear probe |  |  |  |  |
| 1.6.4. Assess spleen with colour Doppler |  |  |  |  |
| 1.6.5. Label all the anatomical structures correctly on the ultrasound image |  |  |  |  |
|  |  |  |  |  |
|  |  |  | **Sub-total** | **/15** |
| **Comment:** | | | | |
| **1.7. Pancreas** |  |  |  |  |
| 1.7.1. Scan the head, neck, body and tail of pancreas |  |  |  |  |
| 1.7.2. Measure the head, neck, body and tail of pancreas |  |  |  |  |
| 1.7.3. Identify the splenic vein, SMA, portal splenic confluence, AO & IVC |  |  |  |  |
| 1.7.4. Label all the anatomical structures correctly on the ultrasound image |  |  |  |  |
| 1.7.5. Compare pancreas and left lobe of liver echogenicity |  |  |  |  |
|  |  |  | **Sub-total** | **/15** |
| **Comment:** | | | | |
| **2. Equipment** |  |  |  |  |
| 2.1. Adjust instrument settings appropriately. TGC, focal zone, frequency, gain setting |  |  |  |  |
| 2.2. Handle the transducers correctly and with ease |  |  |  |  |
| 2.3. Make good use of ultrasound machine |  |  |  |  |
|  |  |  | **Sub-total** | **/9** |
| **Comment:** | | | | |

| **3. Timing** | | | | |
| --- | --- | --- | --- | --- |
|  | Good (3) | Fair  (2) | Poor  (1) | Critical error/ Not done (0 or fail) |
| 3.1 Complete the examination within stipulated time (25 minutes = scanning time) |  |  |  |  |
| 3.2. Work in a logical/methodology manner |  |  |  |  |
|  |  |  | **Sub-total** | **/6** |
| **Comment:** | | | | |
| **Marks** | | |  |  |
| 1. Technique | | |  |  |
| 1.1. Aorta and IVC | | | 18 |  |
| 1.2. Liver | | | 18 |  |
| 1.3. Gallbladder | | | 18 |  |
| 1.4. Right kidney | | | 12 |  |
| 1.5. Left kidney | | | 12 |  |
| 1.6. Spleen | | | 15 |  |
| 1.7. Pancreas | | | 15 |  |
|  | | |  |  |
| 2. Equipment | | | 9 |  |
|  | | |  |  |
| 3. Timing | | | 6 |  |
| **Total** | | | **123** | **/123** |
|  | | |  | **%** |
| **Comment:** | | | | |
| **Examiner name** |  | | | |
| **Examiner signature** |  | | | |
| **Date** |  | | | |
|  |  | | | |
| **Moderator name** |  | | | |
| **Moderator signature** |  | | | |
| **Date** |  | | | |

**** A critical error refers to any action performed by the student sonographer that will cause or lead to misdiagnosis of the patient, or an inconclusive evaluation that would require reevaluation, e.g., mistake the right side for the left or inability to distinguish or correctly identify the anatomy under investigation, ****
